# State Legislation Savvy: A Primer and Tools for Online Legislative Research in the United States

**Published:** 2011-12-15

**Authors:** Leah M. Nguyen, Amy A. Eyler, Jooyoung Kong, Ross C. Brownson

**Affiliations:** Prevention Research Center in St. Louis, George Warren Brown School of Social Work, Washington University in St. Louis; Prevention Research Center in St. Louis, George Warren Brown School of Social Work, Washington University in St. Louis, St. Louis, Missouri; Prevention Research Center in St. Louis, George Warren Brown School of Social Work, Washington University in St. Louis, St. Louis, Missouri; Department of Surgery and Alvin J. Siteman Cancer Center, Washington University School of Medicine, St. Louis, Missouri

## Abstract

We describe sources and methods for state legislative research and provide access to the State Legislative Search Guide tool. State legislation creates and regulates chronic disease prevention interventions both directly through programs targeted to reduce the chronic disease burden and legislation affecting environments such as parks and trails that support health behaviors. Researching state legislation helps advocates, policy makers, researchers, and practitioners make informed recommendations to improve chronic disease prevention policies. Several online sources exist for state legislative information, including subscription databases that cover all 50 US states, single-state subscription databases, and public domain state legislative databases administered by each state. The State Legislative Search Guide, in full-length and condensed versions, uses free public domain databases to facilitate comparison of state legislation for all US states. Links to both versions are provided in the article. Legislative research tips on creating search phrases, searching bill content, bill tracking, and selecting databases and also a table of major subscription databases are provided.

## Introduction

Through the constitutional doctrine of reserved powers, individual states have the authority to protect the public's health (1). Environmental and policy interventions may lower the chronic disease burden at the population level (2-4). State legislation affects multiple areas related to the prevention of chronic disease in school, workplace, and community settings. For example, the regulation of school nutrition and physical education (5-7) and funding of parks (8), trails (9), and farmer's markets (10) make healthy choices easier by affecting access to physical activity and fresh fruits and vegetables.

Researching state legislation is essential to understanding the policy process for advocates and researchers and is facilitated by legislative search engines. Policy researchers use these online databases to identify patterns in state policy trends over time, including trends in state physical education policy (5) and state-to-state differences in childhood obesity prevention policy (11). Advocates can review bills to identify champions and possible hurdles for proposed legislation on their topic of interest. Legislative research can also help 1) reveal the correlates of enactment, such as funding (12) or topic-specific predictors (11) — allowing tailored efforts to increase chances of enactment; 2) establish a schedule for advocacy intervention on the basis of bill tracking and committee hearings; 3) evaluate the relationship between legislative content and population-level outcomes (13); and 4) create policy classification tools like those related to school nutrition environments and physical education (6,7). Consequently, legislative research is the foundation for developing model legislation for public health (14). Model legislation provides sample language to use in the introduction of new legislation. Use of model legislation can help to standardize laws across states and when based on evidence, can help ensure the most effective policies. For example, evaluating the degree to which legislation is evidence-based (13) by scoring each evidence-based criterion for increasing physical activity in children (5) can help to develop policy benchmarks and standards.

In this article, we describe the application of state legislative research for chronic disease prevention. This article focuses on the State Legislative Search Guide tool, which facilitates use of the 50 public domain state legislative databases. We also provide methods and sources to assist policy makers, advocates, practitioners, and researchers in research design and in selecting the most appropriate state legislative database tools for their needs.

## Developing the State Legislative Search Guide Tool

States provide access to searchable databases of legislation, statutes, and other legislative information on their legislature websites. These state-run legislative databases are in the public domain and are free. Our team compared state databases from 6 states to the paid-subscription databases NetScan (15) and LexisNexis (16), which compile legislative information from the 50 states. In the 6 states surveyed, state databases were similar to subscription databases in the number of relevant bills returned in searches. However, the state databases were difficult to use for cross-state legislative research because of the following elements:

- Fifty different Web platforms make navigation time-consuming.
- Database functionality differs from state to state, ranging from rudimentary to advanced.
- Past legislation can be difficult to search for in some states. For example, in Illinois, navigating to past legislative session information is difficult, and New York provides inconsistent access to past legislation.
- The span of legislation that each state database provides varies greatly. Pennsylvania offers legislation back to 1971, while Massachusetts only goes back to 2009.
- The use of *markup language* varies from state to state. Markup language is the coded language (eg, underlined text for newly inserted language and stricken text for deleted language) used to denote proposed changes to existing law or amendments to prior versions of the bill.

To minimize these barriers, we compiled the State Legislative Search Guide of the 50 state legislative websites/databases. The first guide was compiled in 2009 and was updated in 2011 by establishing a matrix of information to collect for each state:

- **Text searches:** A simple search term was selected to test the functionality of the state databases. We chose "physical education" because all states have legislation from recent sessions related to physical education and it is simple enough to test the effect of quotes and *wildcard characters,* placeholders for letters or parts of words (eg, phys* could be used to retrieve physical or physics).
- **Bill number searches:** We searched for a known bill by using its number (eg, HB10 — House Bill 10) to test the functioning of this part of the database. States sometimes refer to searching for a bill by number as *bill tracking*.
- **Contact information:** The Search Guide has a telephone contact for each state, often the legislative librarian.
- **Hyperlinks:** Hyperlinks for the main legislative website, bill search pages, and statute search pages were collected for each state, and hyperlinks to subscription bill tracking pages and legislative glossary pages were collected where they were available.
- **Navigation instructions:** Websites were reviewed and navigation directions were assembled for locating bill search pages, statute search pages, bill tracking services, glossaries of legislative terms, and instructions on how to change sessions while searching.
- **Search tips and general notes:** Each website was reviewed to assemble search tips and any general notes for use.
- **State's legislative staff:** Legislative librarians and webmasters were contacted with questions about new additions, use of markup language in their states, and unique features of their state legislature.
- **Information from the National Conference of State Legislatures (NCSL):** These variables include session length of the legislature, governor's line-item veto power, term limits for legislators, and the type of legislature — an NCSL coding system for full- to part-time legislatures.

The information was compiled in the State Legislative Search Guide (http://prcstl.wustl.edu/Documents/2011%20State%20Legislative%20Search%20Guide.pdf).

We also developed the Condensed State Legislative Search Guide for rapid navigation between state legislative websites and bill search pages (http://prcstl.wustl.edu/Documents/Condensed%202011%20State%20Legislative%20Search%20Guide.pdf). This condensed version includes hyperlinks to the legislature homepages and bill search pages, contact information, and years covered by the legislative database for each state. Both versions of this guide will be updated annually.

## Limitations of the Search Guide Tool

Decisions about what to include in the search guide were made by our research team and were biased according to our research needs for text searches of introduced and enacted legislation in past and current sessions. Our team continues to identify information that could be of use to researchers and advocates that use the guide. We have identified the following limitations that may be addressed in subsequent versions of the guide:

- The District of Columbia is not included.
- There is no information listed on the frequency of updates to the state legislative websites. Our team has been informed that some states lag behind in posting new legislation to their websites, and currency of information is important for policy advocates and lobbyists.
- The Search Guide lists some variations in state database capacity; records of these variations need to be made more consistent and more in-depth. Variation in the capacities of state databases extends to how states set the parameters for text searches of legislation. For example, a text search of the Alaska database searches only the short and long title of the legislation, so a search for legislation with key words "childhood obesity" may not return all of the relevant bills in Alaska.

## Comparing the Different Research Sources for State Legislation

### State databases

Most individual public domain state databases provide a free source of legislative information with the quickest input of each day's legislative action. However, some states (eg, Massachusetts, Ohio) often delay updates (personal oral communication with Ron Hogan, Vice President of CQ State Track, April 7, 2011).

State databases have good searching capacity, although some are limited in their scope. For example, Arizona does not provide advanced search options, such as use of quotation marks to search for precise phrasing in bills; thus, searches may return many irrelevant bills. Massachusetts currently provides only legislation dating back to 2009, although older legislation was previously available. By comparison, Pennsylvania and South Carolina offer searchable legislation dating back to 1971 and 1975, respectively. The search engines used by databases also use different search algorithms; a few are powered by Google (eg, Illinois, Indiana, Maryland, Wyoming). This makes for very different search results than a database that uses *Boolean* search logic. Boolean searching uses AND, OR, NOT and NEAR to limit or expand search results. Although the varying formats and capacities of the state legislative websites can make searching multiple states challenging, comparative research using only the state websites can be conducted. The State Legislative Search Guide tool makes this process considerably less time-consuming.

### Subscription databases

An alternative to the publicly available state databases, subscription databases are easier to use and are less time-consuming for searching multiple states, particularly if large amounts of legislation across multiple states need to be compared or complex topics need to be researched that could benefit from the consistent use of Boolean searching. However, this convenience may be cost prohibitive for individuals or small organizations.

Each subscription database provides slightly different services tailored to meet the needs of their target audience (Table). These subscription databases all obtain their legislative data from the state databases directly or indirectly (LexisNexis and Westlaw obtain their data from State Net [another subscription database, now owned by LexisNexis]) and some contract with private, single-state databases in select states to collect the information at state legislatures (personal oral communication with Ron Hogan, Vice President of CQ State Track, April 7, 2011).

Single-state subscription databases also exist for some states. Many are listed at the website for National Online Legislative Associates (NOLA) (http://www.nolamembers.org/). Most of these single-state databases focus on providing tracking/notification services and documents related to legislation in the current session. These single-state subscription databases can be especially useful when searching for state legislation in states such as Missouri, which lacks both advanced search options and automated bill tracking. Advocacy organizations that focus on lobbying in Missouri may subscribe to GovWatch (http://govwatch.harvestmanager.net/mx/hm.asp?id=home). If the limitations of a state database make it unsuitable for your needs, these single-state subscription databases provide a more affordable option than their 50-state counterparts.

## Legislative Summaries and Topical Databases

Topical databases and legislative summaries are compiled by several different organizations as a resource to advocates, practitioners, policy makers, and researchers in that field. They collect relevant legislation in a topic area and provide summaries of the bills. Usually, these summaries are not available for legislation in the current session but vary widely in their update frequency. A few examples of legislative summaries and databases include the following:

- The Centers for Disease Control and Prevention compiles The Nutrition and Physical Activity Database, which was updated in 2011 to include many new topics and now includes legislation from 2001 through 2011 (http://apps.nccd.cdc.gov/DNPALeg/index.asp).
- The NCSL assembles legislative summaries and databases on topics relevant to chronic disease prevention (http://www.ncsl.org/Default.aspx?TabID=788&tabs=856,34,736#856). This site can be difficult to navigate. Select a collection to view (eg, "Health") at the top of the screen to view the summaries that may be of most interest. Clicking on the "Title" heading sorts the summaries alphabetically to facilitate finding a summary (eg, "Childhood Obesity"). Be sure to bookmark relevant summaries.
- The Yale Rudd Center for Food Policy and Obesity provides a database of current federal and state legislation updated each weekday that is convenient for tracking bills relevant to food policy and obesity (http://www.yaleruddcenter.org/legislation/).
- The American Lung Association's Tobacco Policy Project/State Legislated Actions on Tobacco Issues (SLATI) has compiled summaries of tobacco laws (http://slati.lungusa.org/states.asp).
- The Center for Obesity Prevention and Policy Research (COPPR) created a database of Missouri Health Policies that can be searched by topic, county, region, or policy environment. These policies are at the county rather than state legislative level (http://coppr.wustl.edu/hacpa/data.aspx).

## Legislative Research Tips

### Getting started

A review of literature in your topic area will help you to identify effective search strategies, pertinent language, topics to include, and theoretical frameworks. A scan of the legislative summaries and topical databases relevant to your topic can serve as an introduction to the relevant legislation.

### Creating search phrases

Knowing the particular language used in legislation is essential to designing search phrases that will capture the most relevant legislation. Consider versions of words or phrases that can be used; for example, some bills refer to "Farmers Markets" and others to "Farmer's Markets" or "Farmers' Markets." Scanning the bill summaries listed above will help you to identify specific words to include in your search phrases. The summaries can later provide a comparison for the list of bills returned by your search phrases. If the search phrases do not capture all the relevant bills in the summaries, you will need to either edit or add a new search phrase.

Proper use of wildcard characters (eg, farm* would return farm, farmer, farmers, farmstead, farmed) can increase the power of your search phrase. Wildcard characters (eg, *, %, !) and Boolean syntax varies by database. Operations inside the parentheses are done prior to those outside the parentheses, similar to algebra.

- After School Recreation: ("after school program%" or "summer program%" or "joint use agreement%" or "cooperative partnership%") and ("physical activity" or "physical fitness" or "recreation" or "exercise")
- Farmers Market: ("Farmer's Market Nutrition Program" or "Farmers Market Nutrition Program" or FMNP or F.M.N.P. or "farmers market" or "farmer's market" or "farmers' market")
- Soda Tax: (soda or "soft drink%" or "soft-drink%" or "sugar sweetened beverage" or "sugar-sweetened-beverage" or pop or "bottled water" or "bottled-water") (w/25 [tax*]).

### Reviewing bill content

State databases vary in their methods of demarcating new and deleted language within bills. Some states underline new language and strikethrough deleted language; others use different colors of font, all capital letters, italics, or brackets (ie, {}). States also vary in the meaning of the markup language. For example, in Washington State, markup language always references changes to existing law and does not notate changes between versions of the same bill. However, in California, the same markup language is used to indicate changes to existing law in the introduced version of the bill and amendments in all subsequent versions. Arkansas, Kansas, Maryland, Pennsylvania, and Utah have different markup coding for changes to existing law and bill amendments. Consequently, careful reading of legislation is necessary to determine if the language that you are interested in is in proposed legislation or in already existing law.

Several tools can assist in navigating these differences. The 2011 State Legislative Search Guide details the format and use of markup language for each state to help users identify new, deleted, and amended language in legislation. Some subscription databases reformat the bills so that they have consistent formats; for example, in Lexis Advance, a LexisNexis product, all bills appear with green highlights for added language and red strikethroughs for deleted language. Microsoft Word 2007 has a Compare Documents function under the Review tab (Word 2002 also has this function). This function allows you to view the deletions, new insertions, and movement of text between 2 versions of the same bill. To use this function, copy and paste the versions of the bill that you would like to compare into 2 separate documents. You can find online tutorials to walk you through the steps for comparing documents in Word. These tools can help when you need to compare versions of the same bill or compare proposed legislation to existing law.

When conducting legislative research, think about the provisions of the bill. Look carefully for language that mandates change, provides funding for programs, or requires evaluation or enforcement; all of these elements can increase a bill's potential impact. The Control F (Find) function in your web browser or MS Word and the search function in Adobe PDF are invaluable in helping to quickly locate specific language or wording. Careful reading of the full text of the bill is also important.

### Bill tracking

Bill tracking is the act of following a bill through the legislative process. Policy advocates may be most interested in keeping track of the sponsors, revisions, committee hearings, and votes on particular pieces of current legislation.

Thirty-one states offer free subscription bill-tracking services for current legislation (see the Search Guide). Some states limit the number of bills users can track (eg, Virginia allows 5, Nebraska allows 15, and Oklahoma allows 20), and other states have no limit. Indiana, Kansas, Nevada, and North Dakota offer paid services. In 15 states, all bill tracking must be done manually by conducting bill status searches. The subscription databases Capitol Watch, CQ State Track, and State Net offer bill tracking for all 50 states.

### Selecting databases

Different combinations of database resources fit different needs. Researchers or advocates with limited funds may find legislative summaries and public domain state databases to be their best option. An advocacy group focused on a single state may be best served by a single-state subscription database or their state's public domain database if it offers bill tracking services. Comprehensive scans of legislation across states will save time and ensure consistency by using a 50-state subscription database with select searches in state databases and a review of summarized legislation to check comprehensiveness. If you are considering subscribing to a database, it is advisable to interview representatives from each and ask about your particular needs and how they would be met by the databases. These companies will usually provide free or low-cost trials of their database products for potential customers.

## Conclusion

Searching state legislation online can be a powerful tool for public health policy advocacy and research. Historical research of legislation and its sponsors can provide advocates with a list of possible champions for new legislation and provides opportunities for multiple analyses by researchers. Tracking current legislation allows advocates to identify legislation of interest and stay up to date on committee hearings and amendments. Legislative summaries can act as concise introductions to public health policy topics or checks of search comprehensiveness in other databases. Although subscription databases provide the most user-friendly services, the costs can be prohibitive. State databases usually provide the same information at no charge with the most frequently updated information. The State Legislative Search Guide tool provided in this article can facilitate effective searches of state databases, which vary widely in their formats and capacities.

## Acknowledgments

This article and the development of the State Legislative Search Guide were made possible through funding from the Robert Wood Johnson Foundation (grant no. 65559) for the State Childhood Obesity Policy Evaluation (SCOPE) project and the Centers for Disease Control and Prevention, Prevention Research Centers Program (Cooperative Agreement Number U48DP001903). We thank Kathyrn Barnhart, Jillian Callanan, and Rachel Seiler for research assistance; the legislative librarians and webmasters in the 50 states who answered questions to assist in compiling the search guide tool; and staff at CQ State Track, State Net, Thompson Reuters, and LexisNexis for providing information on their database products for the table.

We thank our colleagues in the Rhode Island Department of Health for their comments and suggestions as the manuscript evolved. We also thank William W. Thompson, PhD, and Kurt Greenlund, PhD, Centers for Disease Control and Prevention, for reviewing and commenting on the final draft. This work and the 2008 Rhode Island Behavioral BRFSS were supported in part by the Chronic Disease Prevention and Health Promotion Programs Cooperative Agreement no. 5U58DP122791-05.

Because the Search Guide will be updated annually, the research team welcomes corrections and suggested additions to improve use.

## Figures and Tables

**Table.** Subscription Databases Providing Access to Legislation for all 50 States^a^

**Characteristic**	Database				

	**Capitol Watch**	**CQ State Track**	**LexisNexis**	State Net	**Westlaw**
**Web address**	http://store.westlaw/general-counsel/capitol-watch/default.aspx	http://www.cqstatetrack.com/	http://www.lexis.com/	http://www.statenet.com	http://store.westlaw.com/westlaw/
**Source of information**	Federal and state bills and other state and federal documents	State databases	State Net, a LexisNexis Company^b^	Multiple data sources (Internet sites, paid state contacts, and online services)	US Library of Congress; State Net, a LexisNexis Company
**Parent company**	Thompson Reuters	CQ Roll Call	Reed Elsevier	Reed Elsevier	Thompson Reuters
**Years covered**	Current session	Current session	1995-present for all states; 1991-1994 for select states	2001-present	1996-present for all states; select states, back to early 1990s.
**Target audience**	Government affairs and relations, lobbyists, corporate compliance general counsel, and corporate communications	Advocacy organizations, lobbyists, law firms, government affairs and relations	Academic researchers; legal professionals	Law firms, academics, consultants, government agencies, companies, legal publishers	Legal professionals, educational institutions, businesses and nonprofits, government agencies
**Strengths**	Fast, flexible search options and easy-to-use interface to identify, monitor, track, and report bills	Small, responsive company; fast searching, tracking of current legislation, Web publishing capacity	Flexible search options, easy-to-use interface for monitoring, tracking, and reporting bills and fast delivery of results	Customized services for topical legislative and regulatory tracking, results can be pushed to subscriber via e-mail	A range of database products, all available on a single easy-to-use platform

a NetScan was not included in this table because it was phased out by Thompson Reuters in November 2010.

b State Net was purchased by LexisNexis in December 2010.
